# Malignant adnexal tumour of the shoulder—a rare case report

**DOI:** 10.1093/jscr/rjab025

**Published:** 2021-04-14

**Authors:** Giridharan Shanmugam, Balamurugan Chidambaram, D Princess Beulah, Vignesh Manimohan

**Affiliations:** Department of General Surgery, Government Stanley Medical College, Tamil Nadu Dr. M.G.R. Medical University, Chennai, Tamil Nadu, India

## Abstract

Malignant adnexal tumours are a very rare and highly aggressive primary skin neoplasms. Among them, malignant hidradenocarcinoma is a particularly aggressive tumour that arises from the intradermal duct of eccrine sweat glands. It more commonly arises *de novo* and rarely from a pre-existing hidradenoma. It is an aggressive tumour with regional lymph nodal and distant visceral metastasis. The prognosis is poor with a 5-year survival rate of 30%. Here, we present a 48-year-old female who came with a swelling over the left shoulder. On examination, it appeared to be chronic sebaceous cyst. The patient underwent wide local excision and the specimen was diagnosed as malignant nodular hidradenocarcinoma. Subsequent re-excision and sentinel lymph node biopsy was performed and margins were found to be microscopically negative for tumour. Based on the available literature wide local excision and sentinel lymph node biopsy appear to be the most common initial treatment plans.

## INTRODUCTION

Skin adnexal tumours are a rare group of tumours for which a lot information is not known. They are broad spectrum of benign and malignant conditions and are classified based on their morphological differentiation towards one of the different types of adnexal structures like hair follicles, sebaceous glands, apocrine and eccrine glands. This makes precise identification and histological characterization difficult. They are also markers for syndromes associated with internal malignancies like Cowden and Muire–Torre syndrome. Malignant adnexal tumours are a very rare and highly aggressive primary skin neoplasms. Among them, malignant hidradenocarcinoma is a particularly aggressive tumour that arises from the intradermal duct of eccrine sweat glands. Hidradenocarcinoma is also often referred to as clear cell eccrine carcinoma, malignant nodular hidradenoma, malignant clear cell acrospiroma, malignant clear cell hidradenoma or primary mucoepidermoid cutaneous carcinoma. The most common sites include the head and neck. It more commonly arises *de novo* and rarely from a pre-existing hidradenoma. It is an aggressive tumour with regional lymph nodal and distant visceral metastasis [1, 2].

## CASE PRESENTATION

**Figure 1:**
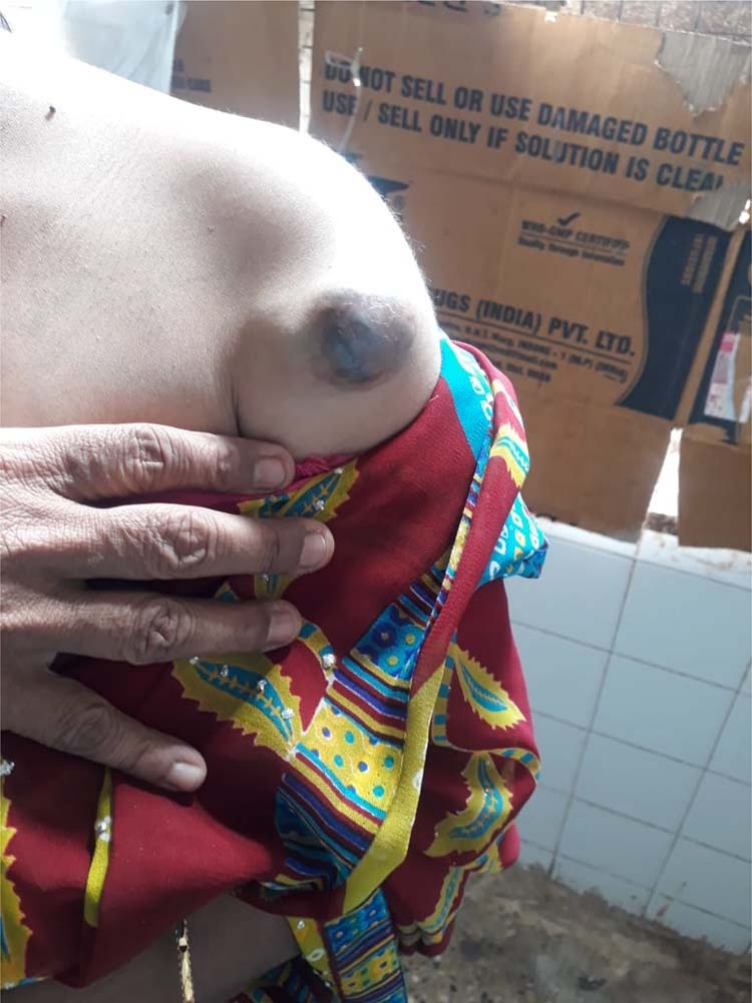
Preoperative image of the lesion.

Here, we present a 48-year-old female without any significant past risk factors or medical history, coming with a 4 × 3 cm well-defined, irregular, non-ulcerated, non-erythematous, non-tender and firm swelling over the left shoulder arising from the superficial plane. Initially, the swelling was smaller in size and was diagnosed as sebaceous cyst by a fine needle aspiration cytology (FNAC) done 3 years back. The patient did not follow up and now reported with complaints of rapid increase in size in the past 6 months. The patient did not have any symptoms and routine physical examination was normal (Fig. 1).

**Figure 2:**
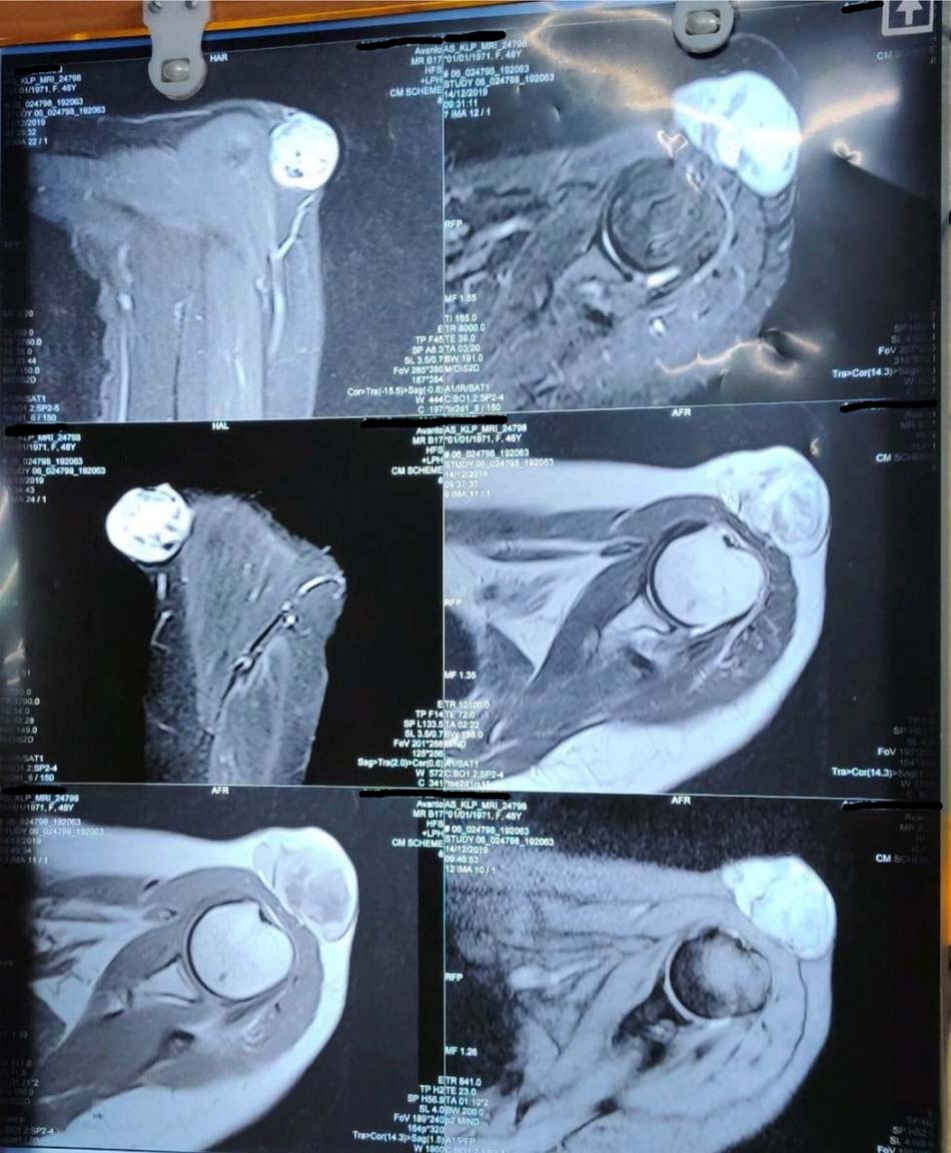
MRI of the shoulder.

The patient was evaluated and magnetic resonance imaging of the left shoulder was done, which showed a solid cystic mass lesion with haemorrhagic areas involving superficial and deep subcutaneous plane with no intramuscular/bony involvement, with the possibility of a soft tissue sarcoma. There was no nodal involvement on radiological imaging (Fig. 2).

**Figure 3:**
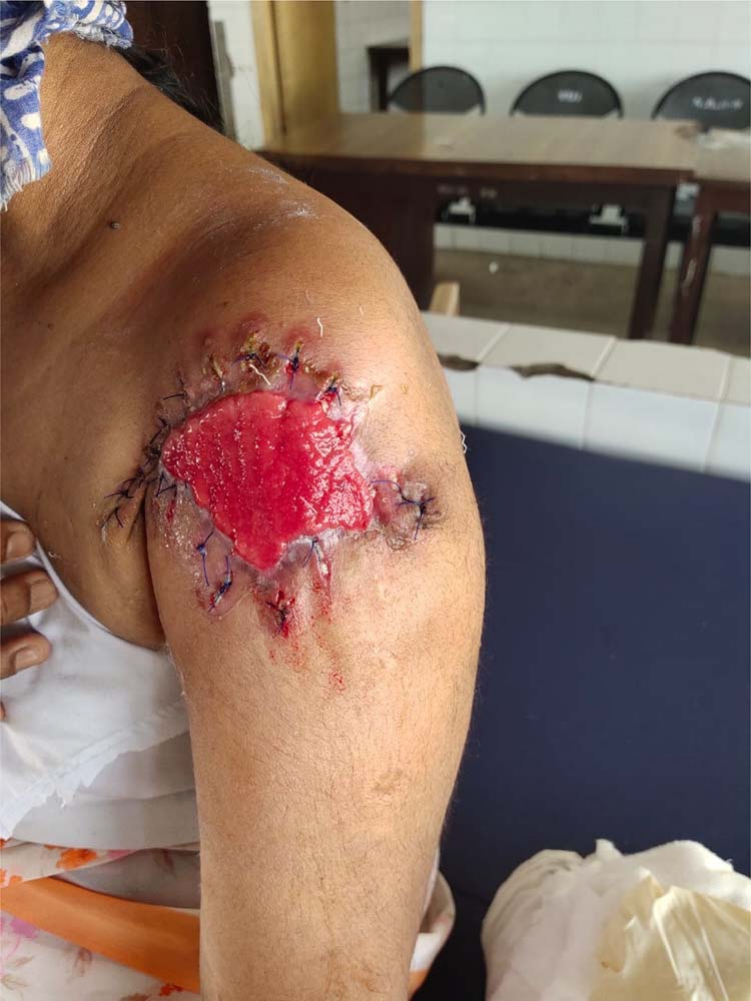
Post wide local excision.

Wide local excision with intraoperative frozen section was carried out. Margins of 1 cm on all side were given clearance and deep margins till pectoralis and deltoid muscle was dissected. Frozen section came out as skin adnexal tumour with tumour-free margins (Fig. 3).

The soft tissue specimen with skin had a cut surface of tumour grey brown in colour with friable necrotic areas. On microscopic examination, the section showed stratified squamous epithelium with an underlying neoplasm composed of lobules of epithelium in a diffuse growth pattern. The epithelial cells show nuclear pleomorphism and are basaloid cells. The tumour also showed closely packed well-developed ducts with extensive areas of necrosis. Few mitotic figures were present in between. Focal areas of necrosis with comedo carcinoma-like appearance were seen. The tumour stained positive for pancytokeratin, cytokeratin 5/6, Ki-67 and S-100. These histological features and stains supported the diagnosis of hidradenocarcinoma.

**Figure 4:**
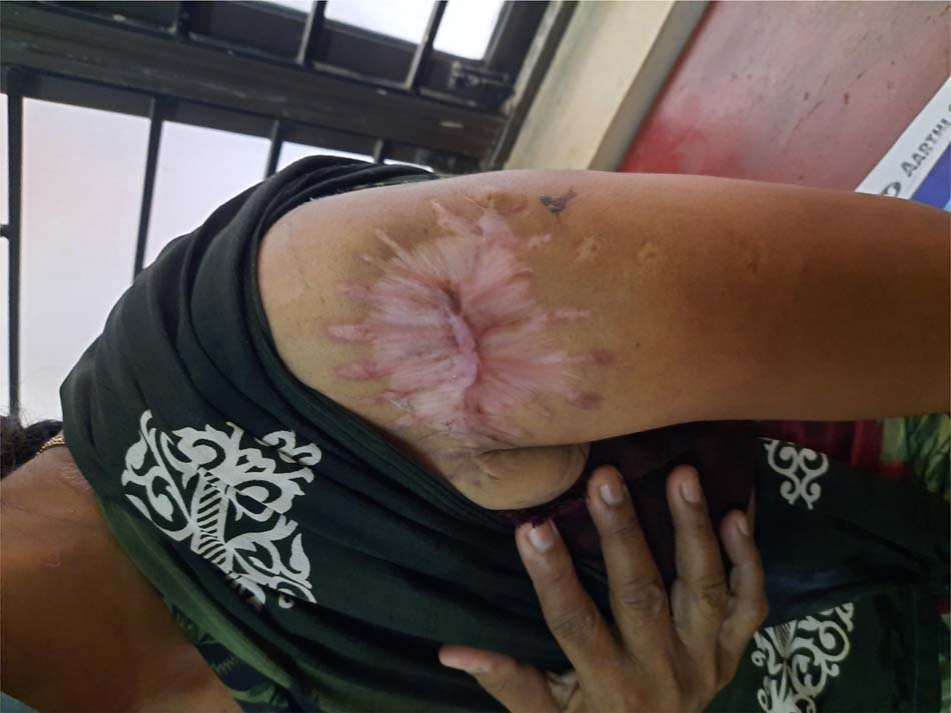
Postoperative scar.

The patient was sent for adjuvant radiotherapy after healing of the surgical site and is currently undergoing radiotherapy (Fig. 4).

## DISCUSSION

Malignant hidradenocarcinoma occurs in the sixth and seventh decades with a slight preponderance among females. However, cases have also been seen in children and neonates. The most common sites include the face, scalp and axilla, but in our case, the tumour was found on the shoulder. It rarely arises from a pre-existing hidradenoma, as in the above case report, and is reported to be *de novo* onset. The most frequent presentation is a nodular mass in the dermis with or without overlying skin changes such as erythema or ulceration. This cancer is typically diagnosed on histopathological characterization as the differential diagnosis is wide, including hemangioma, lipoma, lymphangioma, melanoma, basal cell carcinoma, Merkel cell carcinoma, squamous cell carcinoma and other adnexal carcinomas [3, 4, 5]. Criteria for malignancy include high mitotic activity, atypical nuclei and angiolymphatic or perineural invasion. The immunohistochemistry staining for hidradenocarcinoma is positive for Ki-67, p53, keratin AE1/AE3 and cytokeratin 5/6, but negative for CEA, S-100, GCDFP-15, EMA and bcl-2. Some reports have also found positivity for HER-2/neu and for androgen receptors [6, 7, 8].

## CONCLUSION

Malignant hidradenocarcinoma is a rare tumour with a low incidence rate. There have been only a few reported cases of this cancer in literature and that too arising from a pre-existing hidradenoma on the shoulder region has not been quoted in any of the previous incidences. Further evaluation is required on malignant skin adnexal tumours to assess their response to chemoradiation and formulate effective treatment plans.
